# Colosalpingeal fistula with pneumosalpinx secondary to diverticular disease

**DOI:** 10.1016/j.radcr.2026.06.017

**Published:** 2026-07-07

**Authors:** Fiona Doolan, Fatejeet Sandhu, Francis Flaherty

**Affiliations:** a Zucker School of Medicine, Hofstra/Northwell Residency in Diagnostic Radiology, Norwalk Hospital, Norwalk, CT, USA; b Interventional Radiology, Northwell Health, Danbury Hospital, Danbury, CT, USA

**Keywords:** Colosalpingeal fistula, Salpingoenteric fistula, Tubo-enteric fistula, Diverticulitis, Diverticular abscess

## Abstract

A postmenopausal woman with abdominal pain underwent CT, with the rare finding of pneumosalpinx produced by colosalpingeal fistula from diverticulitis. Though the burden of diverticular disease is increasing globally, salpingoenteric fistulas remain a serious and under-characterized complication, often only discovered during surgery. Identification of pneumosalpinx, a rare specific imaging finding secondary to this uncommon fistula, aids the radiologist in prospective recognition of this entity and expedites definitive treatment.

## Introduction

The burden of diverticulitis is growing, with acute diverticulitis costing the United States alone over 2.6 billion in annual acute care costs and generating an annual total medical expenditure in excess of 6 billion [[1], [2], [3]].

Progression of the disease process with abscess formation, peritonitis, inflammatory fistulization, or bowel obstruction describes complicated diverticulitis and often necessitates surgical intervention [2]. Fistulization in complicated diverticulitis is common between segments of colon, as well as to the bladder and vagina, which are intimately associated structures [4]. Radiologists are well-versed in these complications.

Colosalpingeal (colofallopial) fistula, a subtype of salpingoenteric fistula, is a rare complication of diverticulitis with the potential to cause significant disability. Such fistulae are estimated to occur in 2% of the cases with fistula secondary to diverticular disease [[4], [5], [6]]. Case reports of colosalpingeal fistula complicating diverticulitis have been published almost exclusively in the obstetrics and gynecology literature [6,7]. In several cases, the primary abnormality of fistula or, indeed, diverticulitis was not anticipated preoperatively, resulting in altered surgical intervention [8,9]. Prompt and comprehensive radiologic evaluation has the potential to add significant value. Identification of a primary colon process with secondary adnexal involvement versus a primary adnexal process has significant impact on therapeutic approach.

CT has a pivotal role in the diagnosis of diverticulitis and prospective recognition of secondary adnexal involvement would expedite urgent intervention, enable safer and more comprehensive surgical planning, reduce the need for reintervention, improve outcomes and reduce fertility consequences.

Herein is presented a case of colosalpingeal fistula with the simultaneous findings of gas in the fallopian tube. The latter sign, air in the fallopian tube or pneumosalpinx, has an estimated specificity of 100% for the detection of colosalpingeal fistula in diverticulitis [7]. Familiarity with this sign is thus invaluable to the diagnostician.

A discussion of CT characteristics of the fallopian tube and ovary and pearls and pitfalls in diagnosis of colosalpingeal fistula follows. Key identifying imaging characteristics for the radiologist are illustrated.

## Case presentation

A 58-year-old woman with a past medical history of uncomplicated sigmoid diverticulitis treated medically in the past and remote caesarean section, presented to the emergency department with 3 days of left lower quadrant pain. An imaging and interventional overview of the patient’s hospital course can be reviewed in Fig. 5.

An IV contrast enhanced CT of the abdomen and pelvis in portal venous phase with coronal and sagittal reconstruction was obtained, employing iterative dose reduction technique (CECT). This demonstrated acute perforated sigmoid diverticulitis without abscess. The patient was admitted for IV antibiotics, fluids and pain control, and discharged the next day. However, the patient continued to have gradually increasing pain, and prompted by new fecal urgency, returned to ED 2 days after discharge. On examination, the patient was tender to palpation in the LLQ without rebound, and afebrile with white cell count (WCC) of 10. Portal venous phase CECT of the abdomen and pelvis revealed a 3 cm collection contiguous with inflamed sigmoid diverticula, involving the ovary and a gas-filled serpiginous structure, the left fallopian tube (Fig. 1). This was consistent with perforated sigmoid diverticulitis with abscess and the rare finding of pneumosalpinx. The patient was admitted to surgery.

**Fig. 1:**
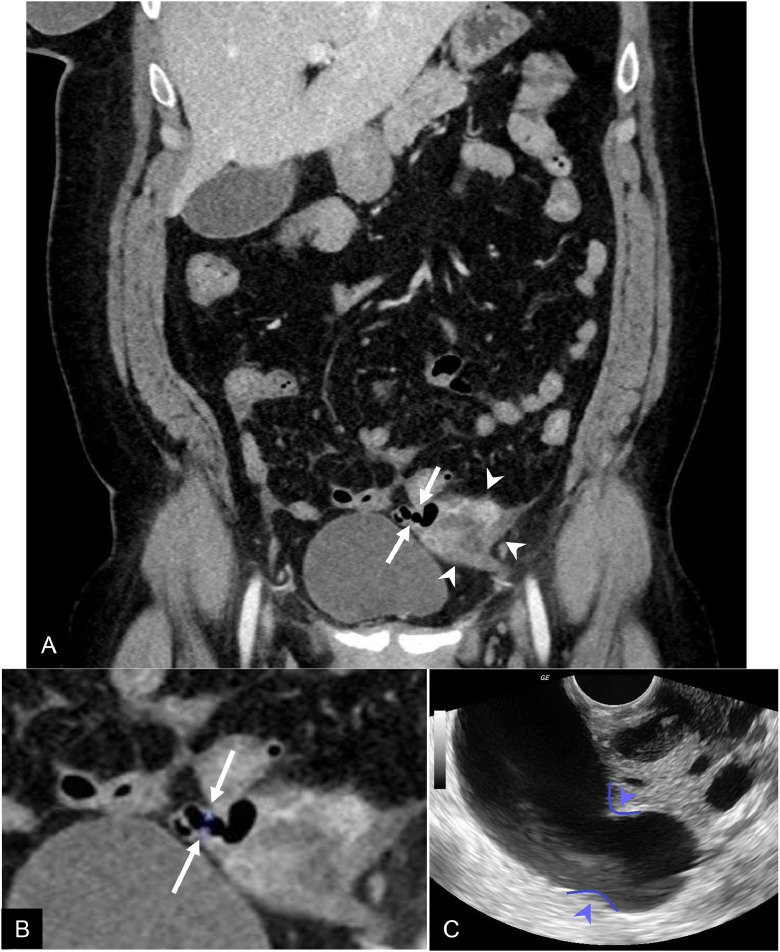
(A) Coronal CECT of the abdomen and pelvis, portal venous phase, 5 mm slice thickness, obtained on admission. Oral contrast administered. There is a gas-filled serpiginous left adnexal structure (white arrows) with 3 cm collection (white arrowheads) contiguous with inflamed sigmoid diverticula. (B) Detail view of the same coronal CECT slice demonstrates waist sign of pneumosalpinx (white arrows). The classical fallopian tube “waist sign” describes the gentle plical indentations of tubal walls of these “C” or “S” shaped structures, and is entirely specific and (C) Example of waist sign in hydrosalpinx on transvaginal ultrasound (blue arrowheads).

Interventional radiology was enlisted to drain the collection on the day of admission. Nonenhanced CT biopsy was conducted. A narrow window was identified between the broad ligament ovarian vessels and the iliac artery and vein was identified, whereupon an 18-gauge Chiba needle advanced inferior to the broad ligament until the needle tip was within the collection and 10 cc purulent material was aspirated. Passed via wire, a 10 French cope loop drainage catheter was placed in the collection with near complete resolution (Fig. 3).

IV broad spectrum antibiotics were continued. WCC trended down over subsequent days and patient tolerated a diet, but began to require increasing pain control, prompting CECT of the abdomen and pelvis on day 5 of admission. This revealed resolution of the drained abscess but interval progression of multiple abscesses in the region of the sigmoid and mesentery (Fig. 4). Monitoring continued.

Due to persistent elevated WCC, unremitting pain and progression of collections, patient was taken to OR day 6 following admission. Robotic sigmoid colectomy, end colostomy (Hartmann's procedure), and drainage of intra-abdominal abscesses was performed by general surgery. Hysterectomy and bilateral salpingo-oophorectomy (THBSO) was performed by gynecologic surgery. Intraoperatively the left adnexa was noted to be adherent to a pelvic collection and to a portion of sigmoid colon, with fistulous communication. IR drain was removed. A Blake drain was placed in the largest collection in the right lower quadrant and removed before discharge following an unremarkable postoperative course. OR wound cultures obtained on antibiotics were positive for candida albicans and fluconazole was added to the outpatient regimen.

Surgical pathology confirmed fallopian tubes showing florid bilateral salpingitis with abscess, and fibrinous adhesions with pyogenic material (Fig. 6).

Robotic assisted colostomy reversal (reversal of Hartmann's) was performed successfully 4 months following the surgery.

## Discussion

This is a rare case of colosalpingeal fistula with pneumosalpinx secondary to diverticular disease. The mechanism is severe inflammation from a perforated diverticulum causing adherence of the bowel wall to the adjacent fallopian tube, leading to tissue necrosis, erosion, and the creation of a direct conduit between the colon and the fallopian tube.

### Anatomy and physiology considerations

The fallopian tube is a thin, ∼10 cm fibromuscular structure extending from the uterus to the ovary, divided into 4 segments: intramural, located in the uterine wall; isthmus, ampulla and the fimbriated infundibulum near the ovary [10].

Medially, the fallopian tube inserts into the uterine cornua at its proximal (interstitial) portion. It is continuous with mesosalpinx, mesentery that carries vessels from the uterine and gonadal arteries. Typically, laterally and inferiorly, the ovary is closely approximated with the fimbriated end of the fallopian tube. Unlike most organ systems, the fallopian tube opens directly into the peritoneal cavity at it fimbriated end and thus provides a direct route for tubal involvement by abscess [11]. The bladder lies anterior to the uterus while posteriorly the rectum and sigmoid colon are interposed behind and around the adnexal structures. The subperitoneal mesometrium (part of the uterovaginal compartment) extends dorsally with suspensory and fatty tissue that fuses to the anterior and lateral mesorectum. Colosalpingeal fistula occurs in the left fallopian tube in 93% of cases, while the right tube is affected in only 7% of cases, due to diverticulitis vastly favoring the left and sigmoid colon [7]. This should be considered in evaluation.

The fallopian tube wall consists of 3 main anatomical layers, the outer serosa, muscularis, and inner mucosa.

The serosa is a thin layer of connective tissue. The muscularis layer consist of multioriented smooth muscle bundles producing a complex muscular network.

The mucosa contains the epithelium and lamina propria. The mucosa has many folds, called plicae, most numerous in the ampulla, where most fertilization occurs. After menopause, the lining contracts and the epithelial cells decrease in number, blunting the plicae [11].

### Identifying colosalpingeal fistula

In CECT with diverticulitis, particularly when complicated by stranding, pericolonic phlegmon, free fluid or abscess, it can be challenging to identify the adnexal structures or ascertain their involvement, even with robust knowledge of locoregional anatomy reviewed above.

In the normal female pelvis, the fallopian tube is usually nearly invisible, containing minimal endotubal fluid and never gas, and is seamlessly incorporated with the mesosalpinx component of the broad ligament along with the ovarian and uterine vessels. When examining for adnexal involvement, a useful first operating principle is to identify the ovarian vein, on the left, joining the renal vein, and on the right, joining the IVC. The ovarian vein is usually prominent excepting in profound hypovolemia, and can be traced to its origin at the ovary [10]. The ovary can be recognized as an approximately 3 cm soft tissue mass with internal fluid density structures, the follicles. Closely associated will be the fallopian tube. This can be particularly helpful in the context of gross ascites, which reduces natural contrast between adnexal structures normally outlined by fat planes.

A pericolonic abscess may look remarkably similar to a colosalpingeal fistula, with both demonstrating enhancing walls and containing simple or complex fluid or gas. When it is difficult to ascertain the borders, knowledge of tubal anatomy can be instrumental. The mucosal plicae are appreciable in fluid (or in this case, air-) -containing fallopian tubes on CECT as incomplete enhancing septations or invaginations (Fig. 1). The classical sign of a dilated fallopian tube is the “waist sign,” describing the gentle plical indentations of tubal walls of these “C” or “S” shaped structures, which is entirely specific [12]. Plicae are most abundant in the ampulla. However, their numbers decrease after menopause or due to scarring from PID and endometriosis, which can obscure the “waist sign.” Abscesses, on the other hand, have complete septations, and do not exhibit waist sign.

An additional sign of a dilated fallopian tube is the “cogwheel” sign (thickened plicae in cross-section), but may be less visible in postmenopausal patients due to aforementioned involutional changes.

**Fig. 2:**
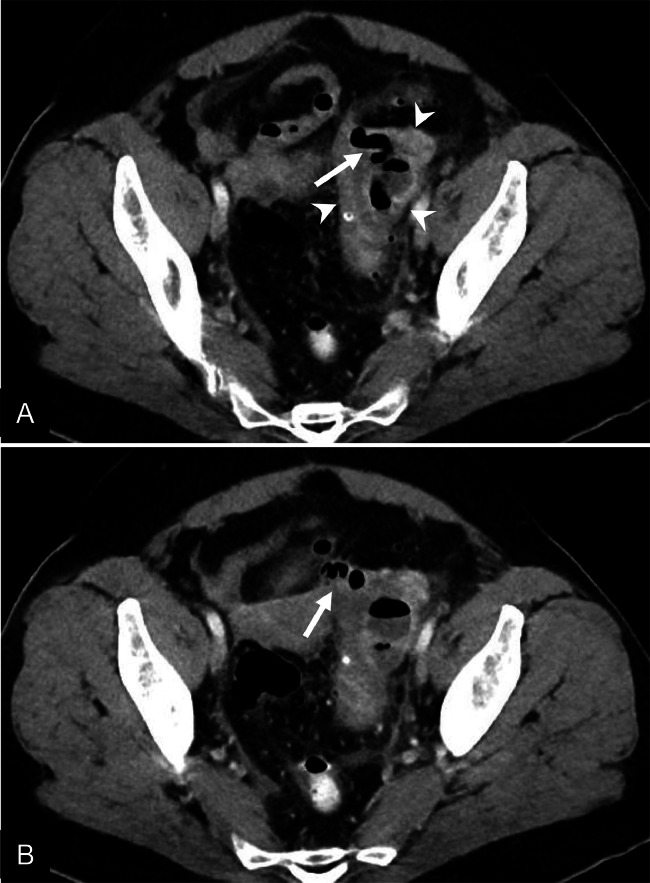
Consecutive axial CECT images in the lower pelvis, portal venous phase, 5 mm slice thickness, obtained on admission. Oral contrast administered. (A) More cranially, the 3 cm enhancing collection with gas-fluid level is interposed among sigmoid diverticular loops (white arrowheads) and contiguous with the air-filled “S” shaped left fallopian tube (white arrow) and (B) Caudal image captures characteristic distinctive serpiginous appearance (white arrow).

**Fig. 3:**
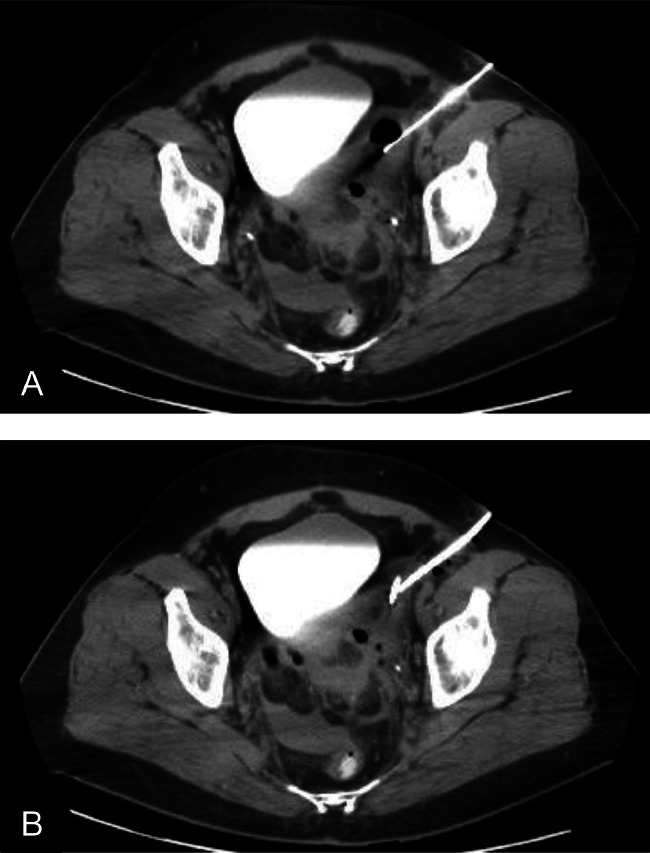
Nonenhanced axial targeted CT for visceral drain placement (excretory phase of IV contrast from earlier same-day CECT). (A) Chiba needle advanced via anterior approach into 3 cm collection containing gas and fluid and (B) Cope loop drainage catheter in place with reduction in size of collection.

**Fig. 4:**
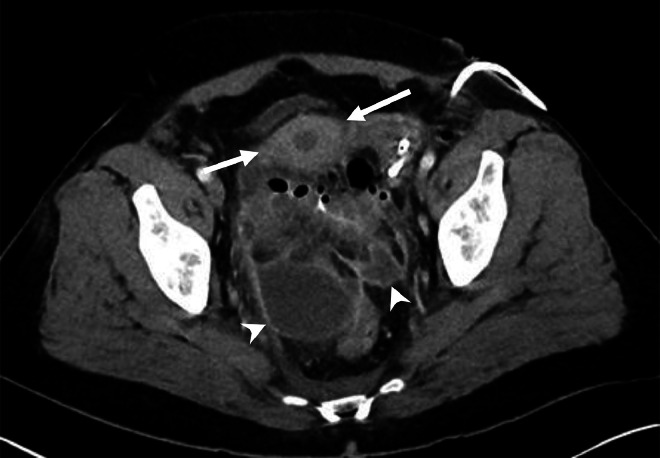
Admission day 5, Axial CECT in portal venous phase. Cope drainage catheter in situ. Within the pelvis adjacent to the right side of the rectum, there is a 4 × 6 cm abscess and on the left side of the pelvis there is a 2.4 cm abscess (white arrowheads). Uterus–white arrows.

**Fig. 5:**
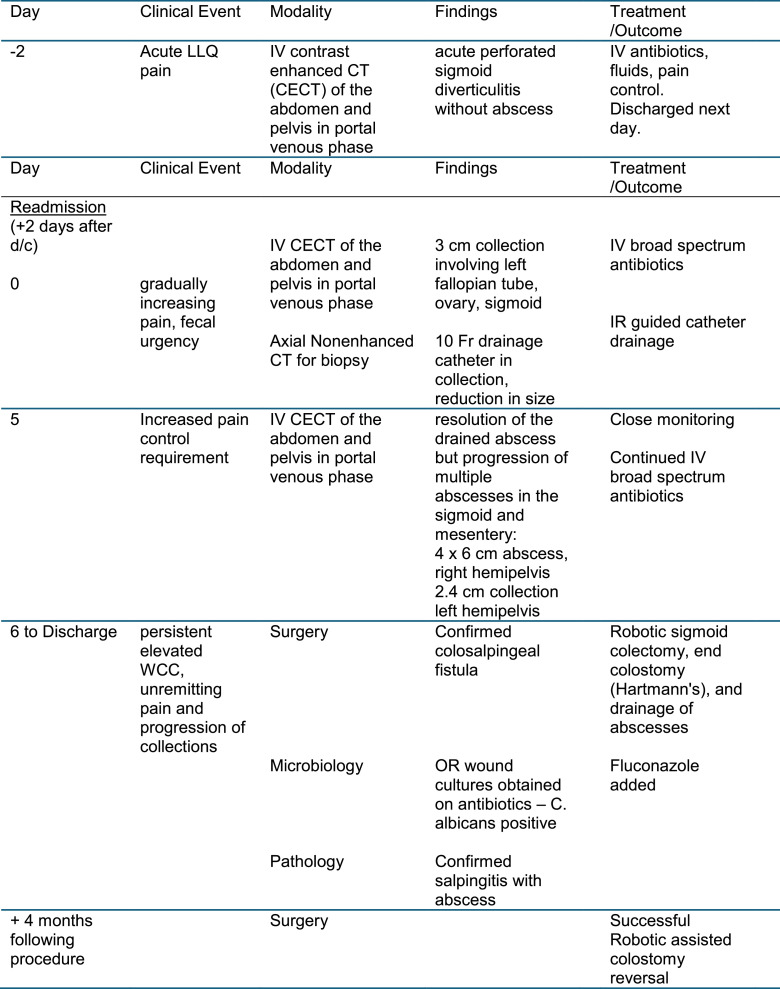
Patient clinical course, imaging, and interventions.

**Fig. 6:**
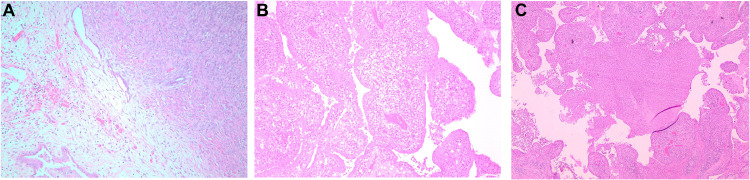
(A) H&E 100x showing cuboidal cells of left ovarian surface epithelium, with superficial fissuring attributable to abscess. Increased neutrophils are reactive. Some intact luteinized ovarian stromal cells with a whorled appearance, polygonal with abundant eosinophilic to clear cytoplasm, occupy the upper right of the slide. (B) H&E 100x of the left fallopian tube lumen demonstrating inflammatory neutrophils and edema within the plicae confirming salpingitis. In this postmenopausal patient, there is expected stromal thinning and (C) Fallopian tube confirming abscess.

Pericolonic abscess, furthermore, will usually have identifiable convex borders surrounded by inflammation, while fallopian tube, unless torsed, is tubular and not enclosed on all sides.

Secondary signs of colosalpingeal fistula include extraluminal gas near the colon, adnexal enlargement, and pneumosalpinx (Fig. 2). Pelvic free fluid, though nonspecific, warrants closer inspection for this entity.

If further imaging is warranted before a decision for surgical intervention, fluoroscopic hysterosalpingogram is a key confirmatory dynamic study for colosalpingeal fistula. The diagnosis can be confidently made without additional imaging, however, when pneumosalpinx is identified.

## Conclusion

Diverticulitis with extensive complications, including fistulae and abscesses, may often present in an indolent manner, resulting in undertreatment by conservative management where surgery is indeed required [6,7]. One proposed mechanism is effective sequestration of collections by a robust body response, which prevents the development of fulminant peritonitis. In such cases the radiologist plays the key role in defining extent of disease, and thus clinical course. This could mean the difference between urgent, definitive surgical treatment and delay in care. Prospective recognition of this gynecologic complication of diverticulitis is extremely clinically relevant, as untreated or undertreated fistulas can lead to serious complications, including infections, tubal damage and infertility, and repeated surgery. Prompt diagnosis of colosalpingeal fistula can often enable fertility conserving treatments. This is especially prescient, as the incidence of diverticular disease is increasing in younger populations [13]. Radiologists are encouraged to maintain a high index of suspicion for adnexal involvement in perforated diverticulitis.

This case of colosalpingeal fistula in complicated diverticulitis is an example of a rare complication of a commonly encountered disease. Unusual presentations of common diseases are overrepresented in actual clinical practice, but rarely emphasized in medical literature. We hope this case provides some practical insight into the matter and underscores the importance of considering uncommon complications of routine pathologies in radiologic evaluation of the acute abdomen.

## Patient consent

A written informed consent was obtained from the patient for the publication of this case report.

## References

[1] Peery A.F., Crockett S.D., Barritt A.S., Dellon E.S., Eluri S., Gangarosa L.M. (2015). Burden of gastrointestinal, liver, and pancreatic diseases in the United States. Gastroenterology.

[2] Carr S., Velasco A.L. (2025). StatPearls.

[3] Brown R.F., Lopez K., Smith C.B., Charles A. (2025). Diverticulitis: a review. JAMA.

[4] Strate L.L., Morris A.M. (2019). Epidemiology, pathophysiology, and treatment of diverticulitis. Gastroenterology.

[5] Rose J., Parina R., Faiz O., Chang D., Talamini M. (2015). Long-term outcomes after initial presentation of diverticulitis. Ann Surg.

[6] Tancer M.L., Veridiano N.P. (1996). Genital fistulas secondary to diverticular disease of the colon: a review. Obstet Gynecol Surv.

[7] Panghaal V., Chernyak V., Patlas M., Rozenblit A. (2012). CT features of adnexal involvement in patients with diverticulitis. Am J Roentgenol.

[8] Bourgan L.M., Emole A., Khalil M.E., Londree J., Frezza E. (2024). Colo-salpingeal fistula: a rare gynecologic manifestation of diverticular disease. Arch Gynecol Obstet.

[9] Jangam A., Gillespie C. (2022). A rare case of colo-salpingeal fistula complicating acute sigmoid diverticulitis. J Surg Case Rep..

[10] Revzin M.V., Moshiri M., Katz D.S., Pellerito J.S., Mankowski Gettle L., Menias C.O. (2020). Imaging evaluation of fallopian tubes and related disease: a primer for radiologists. Radiographics.

[11] Metcalf B.D., Turashvili G. (2024).

[12] Visnyaiová K., Varga I., Feitscherová C., Pavlíková L., Záhumenský J., Mikušová R. (2024). Morphology of the immune cells in the wall of the human uterine tube and their possible impact on reproduction-uterine tube as a possible immune privileged organ. Front Cell Dev Biol.

[13] Kim S., Kwon O., Chervu N., Mallick S., Ali K., Benharash P. (2025). National trends in hospital admissions, interventions, and outcomes for early-onset (Age <50 years) diverticulitis from 2005 to 2020. Dis Colon Rectum.

